# *Poncirus trifoliata* (L.) Raf. Seed Extract Induces Cell Cycle Arrest and Apoptosis in the Androgen Receptor Positive LNCaP Prostate Cancer Cells

**DOI:** 10.3390/ijms242216351

**Published:** 2023-11-15

**Authors:** Francesca Giordano, Stefano Comità, Giulia Venneri, Vittoria Rago, Giuseppina Daniela Naimo, Francesca De Amicis, Anna De Bartolo, Rosa Tundis, Loredana Mauro, Maria Luisa Panno

**Affiliations:** 1 Department of Pharmacy, Health and Nutritional Sciences, University of Calabria, 87036 Rende, Italy; stefcom85@gmail.com (S.C.); giuliavenneri.311@gmail.com (G.V.); vittoria.rago@unical.it (V.R.); giuseppinadaniela.naimo@unical.it (G.D.N.); francesca.deamicis@unical.it (F.D.A.); rosa.tundis@unical.it (R.T.); mluisa.panno@unical.it (M.L.P.); 2 Department of Biology, Ecology and Earth Sciences, University of Calabria, 87036 Rende, Italy; anna.de_bartolo@unical.it

**Keywords:** LNCaP, *Poncirus trifoliata* seed extract, apoptosis, AKT/mTOR pathway, MAPKs

## Abstract

Prostate cancer (PCa) is the second most common male cancer. Its incidence derives from the interaction between modifiable and non-modifiable factors. The progression of prostate cancer into a more aggressive phenotype is associated with chronic inflammation and increased ROS production. For their biological properties, some phytochemicals from fruits and vegetable emerge as a promise strategy for cancer progression delay. These bioactive compounds are found in the highest amounts in peels and seeds. *Poncirus trifoliata* (L.) Raf. (*PT*) has been widely used in traditional medicine and retains anti-inflammatory, anti-bacterial, and anticancer effects. The seeds of *P. trifoliata* were exhaustively extracted by maceration with methanol as the solvent. The cell proliferation rate was performed by MTT and flow cytometry, while the apoptosis signals were analyzed by Western blotting and TUNEL assay. *P. trifoliata* seed extract reduced LNCaP and PC3 cell viability and induced cell cycle arrest at the G0/G1phase and apoptosis. In addition, a reduction in the AKT/mTOR pathway has been observed together with the up-regulation of stress-activated MAPK (p38 and c-Jun N-terminal kinase). Based on the study, the anti-growth effects of *PT* seed extract on prostate tumor cells give indications on the potential of the phytochemical drug for the treatment of this type of cancer. However, future in-depth studies are necessary to identify which components are mainly responsible for the anti-neoplastic response.

## 1. Introduction

In the most developed countries, prostate cancer (PCa) represents the frequent tumor in men between the ages of 45 and 60 [1,2]. Prostate tumor is a multifactorial disease, whose incidence is the result of a rather complex interaction between genetics and environmental risk factors. Therefore, it is possible to distinguish non-modifiable factors, such as ethnicity, age and hormonal status, and modifiable risk factors, like smoking, overweight, obesity, sedentary lifestyle and poor quality of diet [3,4].

Interactions between genetic and environmental factors cause a deregulation of genes that control epigenetic processes and are involved in histone modifications, DNA methylation and non-coding miRNA [5]. In the epithelial and stromal portions of the prostate gland, testosterone (T) and its derivative, dihydrotestosterone (DHT), which has stronger biological activity, by binding to the androgen receptor (AR) [6] control cell proliferation, differentiation and metabolic-secretory functions of the epithelial tissue. Instead, at the stromal level, the hormones regulate the production of many growth factors, such as the Epidermal Growth Factor (EGF), Fibroblast Growth Factor (FGF), Insulin Growth Factor (IGF), Neuropeptide Growth Factor (NGF), and Keratinocite Growth Factor (KGF), which act in a paracrine way on the epithelial layer, controlling its proliferation and protecting secretory luminal cells from apoptosis [7].

Although normal development of the gland is controlled by androgens, the progression of prostate cancer can be both androgen-dependent and androgen-independent. This distinction is important for the type of treatment, grade, stage and possible recurrence of tumor [8]. In cases of less aggressive prostate cancer, active surveillance is the recommended approach, while androgen deprivation, surgery and radiation are the curative treatments for localized neoplasms even if they have negative side effects on the urinary and sexual activities that affect also the quality of life. For metastatic disease, in addition to androgen deprivation, the treatment of choice includes chemotherapy; both approaches appear to prolong survival compared to single treatment.

Unfortunately, prostate cancer changes over time to become resistant to therapies. In fact, most of the patients have mutated androgen receptors, constitutively active androgen receptor variants and AR gene amplification [9,10,11,12,13]. Furthermore, one of the long-term effects of hormone refractoriness is the onset of the neuroendocrine phenotype that coincides with poor prognosis and the lack of effective strategies against tumors [14].

For this reason, it is important to search for new therapeutic agents with fewer side effects and higher efficacy to complement traditional treatment methods for prostate cancer.

Numerous studies have shown that diet is an important factor in the environmental protection against cardiovascular and neoplastic diseases. In particular, the consumption of fruit in the diet is associated with the prevention and/or protection of various cancer types, such as breast, colon, pancreatic, lung and prostate cancers [15,16].

The Rutaceae family, which includes genera such as *Citrus* and *Poncirus* of great interest from both nutritional and health points of view, is a rich source of flavonoids, limonoids, phenolic acids, and vitamin C [17,18], which retain antioxidant and anti-inflammatory properties, thus playing a key role in cancer chemoprevention.

*Poncirus trifoliata* (L.) Raf, known as “trifoliate orange”, is a deciduous or semi-deciduous plant that grows to over 8 m in height bearing green or yellow fruits resembling small oranges 3–4 cm in size. *P. trifoliata*, which originated in China and Korea, is widely used in traditional medicine for the treatment of some gastrointestinal diseases such as ulcers and gastritis but also to counteract inflammation and allergies [19]. Various biological activities of *P. trifoliata*, mainly including anti-inflammatory [20], anti-bacterial [21], anti-anaphylactic [22,23], and hypoglycemic activities [18] have been described. *P. trifoliata* is also known for its anticancer properties that are related to its main phytochemical compounds, such as phenolic acids, flavonoids, coumarins, alkaloids, triterpenoids and sterols [19,24,25,26,27,28].

To the best of our knowledge, the present study evaluated for the first time the anticancer effects of *P. trifoliata* seeds extract on human prostate cancer cells, using the AR-positive LNCaP cell line and established the underlying molecular mechanisms related to this effect.

## 2. Results

### 2.1. Chemical Profile of P. trifoliata Seed Extract

The seeds of *P. trifoliata* were exhaustively extracted by maceration by using methanol as the solvent. Then, the extract was chemically investigated by using high-performance liquid chromatography–diode array detection (HPLC-DAD). As previously reported, three flavanones such as naringin, neohesperedin, and narirutin, and the phenolic compound caffeic acid were identified as the main compounds. Quantitative analyses (Table 1) evidenced the presence of naringin (156.42 µg/g extract) as the most abundant constituent followed by neohesperedin (80.12 µg/g extract), narirutin (37.62 µg/g extract) and the phenolic compound caffeic acid (32.85 µg/g extract) [18].

### 2.2. Effects of P. trifoliata on Proliferation Rate and Morphological Changes of LNCaP Cells

**Figure 1:**
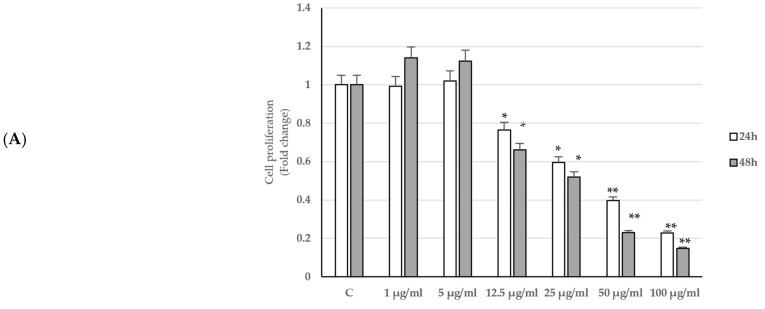

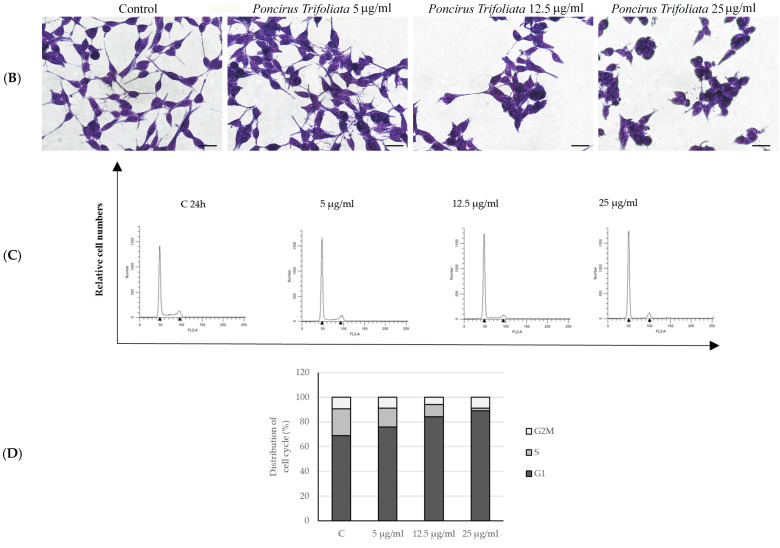
*PT* seed extracts-induced inhibition of proliferation, changes in cell morphology and arrest of the cell cycle. (**A**) LNCaP cells were treated with increasing doses (1, 5, 12.5, 25, 50 and 100 µg/mL) of *PT* seed extracts for 24 and 48 h. The results were expressed as fold change ± S.D. relative to control (C). The histogram is representative of three different experiments, each performed in triplicate. * *p* < 0.05 and ** *p* < 0.001. (**B**) May–Grunwald–Giemsa was used to stain LNCaP-treated cells for 24 h (original magnification, ×200). (**C**) Flow cytometer analysis was used to evaluate the cell cycle after treatment of LNCaP cells with *PT* seed extract for 24 h. (**D**) The histogram represents the percentage distribution of cells in the different phases of the cell cycle.

The effects of *P. trifoliata* seed extract (*PT*) on cell viability was herein evaluated using as an experimental model the LNCaP androgen-sensitive human prostate adenocarcinoma cells. For this purpose, the effects of increasing concentrations (1, 5, 12.5, 25, 50, 100 µg/mL) of *P. trifoliata* seed extract were tested on cell viability by MTT assay after 24 and 48 h of treatment. The treatment with the extract caused a reduction in LNCaP cell viability at both times starting from the concentration of 12.5 µg/mL (Figure 1A). The half-maximal inhibitory concentration (IC_50_) values for the *P. trifoliata* seed extract are shown in Table S1. Staining with May–Grunwald–Giemsa showed morphological changes, such as number and cell volume reduction, as well as a loss of cytoplasmic extensions, which was more evident in the cells treated with 25 µg/mL of *P. trifoliata* extract for 24 h (Figure 1B).

The analysis of cell cycle through flow cytometry revealed that in cells treated for 24 h with different concentrations of *P. trifoliata* extract (5, 12.5 and 25 µg/mL), the percentage of cells in the G1 phase gradually increased by 84.14% and 89.14% when subjected to 12.5 and 25 µg/mL of the extract, respectively, compared to control (68.93%), while in the same experimental conditions, a concomitant reduction in cells in the S phase was evidenced (C: 21.78%; 12.5 µg/mL: 10.07%; 25 µg/mL: 2.02%) (Figure 1C,D).

Similar results regarding cell proliferation and the cell cycle were obtained in an androgen-independent prostate carcinoma PC3 cell line. In fact, we observed a consistent reduction in cell proliferation accompanied by a decrease in S phase and an increase in the percentage of cells in the G1 phase in cells treated with 25 µg/mL of *P. Trifoliata* seed extract (Figure S1A–C).

**Figure 2:**
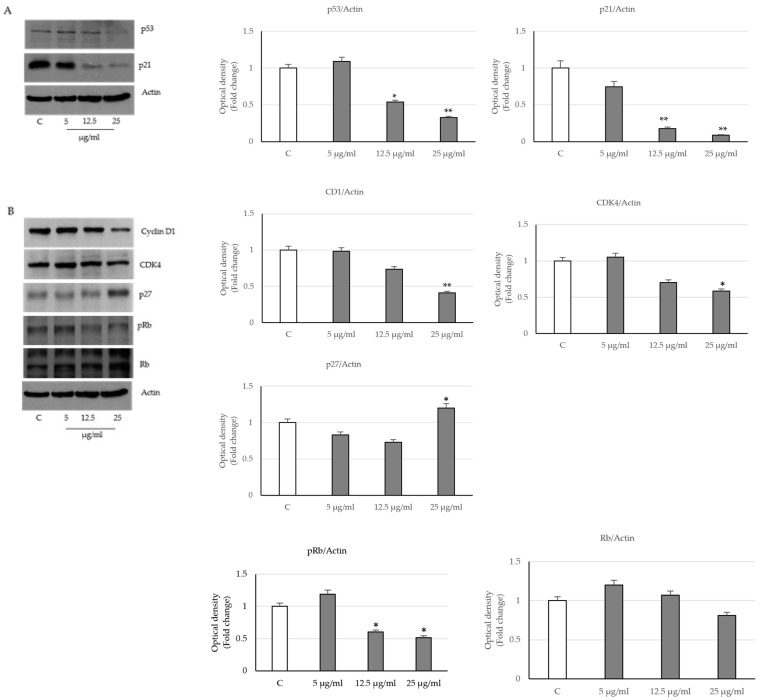
Effects of *P. trifoliata* seed extract on the expression of cell cycle-regulating proteins. (**A**) p53 and p21 proteins were analyzed by immunoblot in LNCaP cells untreated or treated with extracts for 24 h. For the loading control, we used the actin protein. (**B**) The expression levels of cyclin D1, CDK4, p27, pRb and Rb were detected by immunoblot in LNCaP cells. Histograms represent the mean SD of three experiments in which band intensities were evaluated in terms of arbitrary units of optical density and expressed as fold change relative to control. * *p* < 0.05 and ** *p* < 0.001 vs. control (C).

In order to elucidate the detailed mechanisms by which the extract induced cell cycle arrest, we evaluated the expression levels of the well-known protein regulators of cell cycle, such as the tumor suppressor p53, the cyclin D1/CDK4, the CDK inhibitors, p21^waf/cip1^ and p27^kip1^. Western blot analysis showed a significative reduction in the p53 and p21^Cip1/WAF1^ expression levels in prostatic cells treated with 12.5 and 25 µg/mL of seed extract for 24 h compared to control (Figure 2A).

Furthermore, a reduction in the expression levels of both cyclin D1 and the cyclin-dependent kinase, CDK4, was revealed in the same experimental conditions, while a significant up-regulation of the inhibitor of cyclin-dependent kinases p27^kip1^ was evidenced at the highest dose of the extract (Figure 2B).

To assess whether these changes also involve the oncosuppressor pRb protein, we evaluated its phosphorylation status on which its functional activity depends. The immunoblotting analysis revealed a reduction in pRb protein levels in prostatic cells treated with *PT* extracts compared to the control, which started at the dose of 12.5 µg/mL in the presence of unchanged protein levels (Figure 2B). These data support the concept that cell viability repressed by the seed extracts of *PT* may be due to cell cycle arrest that involves the decrease in cyclin D1, the up-regulation of p27 and of the oncosuppressor pRb, which in its hypo-phosphorylated state is active, and in this way, it acts as a brake for the transition of cell cycle toward the S phase.

**Figure 3:**
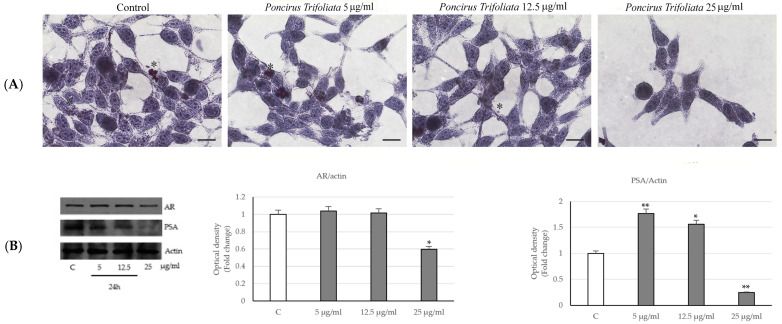
Effects of *P. trifoliata* seed extract on the lipid droplet and on the expression of androgen receptor (AR) and prostate-specific antigen (PSA) proteins. (**A**) Cells were treated with various concentrations of *PT* extracts for 24 h and stained with Oil Red O (scale bars 12.5 µm). (**B**) Immunoblot of AR and PSA expression levels in LNCaP cells at 24 h. Data are expressed as mean ± SD of three experiments. The histograms represent the fold change in quantified value versus control normalized for actin. * *p* < 0.05 and ** *p* < 0.001 vs. Control (C).

Moreover, under the same experimental conditions, cell staining with Oil Red O showed a decrease in lipid droplets especially in samples submitted to the higher doses of treatment. This indicates a drastic reduction in adipogenesis and therefore consequently a decrease in the energy component of the cells (Figure 3A).

Since the androgen receptor (AR) plays an important role in prostatic carcinogenesis, we determined its expression level in LNCaP-treated cells with *P. trifoliata* extract for 24 h. Data revealed a decrease in AR in the cells subjected to the highest concentration of the extract together with the reduction in its dependent PSA protein (Figure 3B).

### 2.3. Activation of the Intrinsic Apoptosis Pathway following Seed Extract Treatment

**Figure 4:**
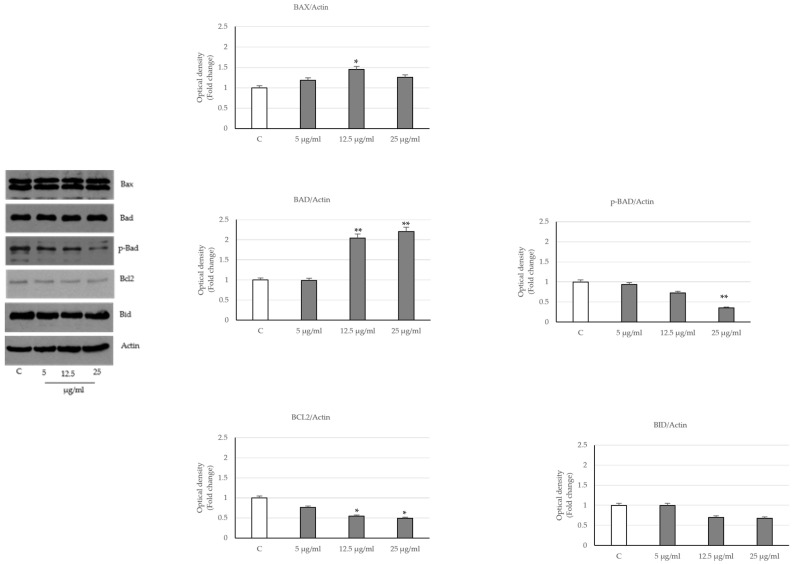
Effects of *P. trifoliata* seed extract on the expression of apoptosis proteins. Immunoblot of Bax, Bad, Bcl2, Bid and p-Bad (ser 112) expression levels in LNCaP cells untreated or treated with seed extracts for 24 h. The optical density data are the mean ± SD of three experiments. * *p* < 0.05 and ** *p* < 0.001 vs. control (C) The loading control was completed by actin.

Then, to explore whether the PT extract induced prostate cancer apoptosis, we analyzed the expression levels of the key proteins that regulate the intrinsic pathway. As shown in Figure 4, in LNCaP-treated cells, we observed an increased expression of the pro-apoptotic proteins Bax and Bad, which are associated with decreased levels of Bcl2 and Bid. In addition, Bad phosphorylation on ser 112, necessary to promote cell survival, results in a reduction in the presence of the phytocomplex compared to control cells.

**Figure 5:**
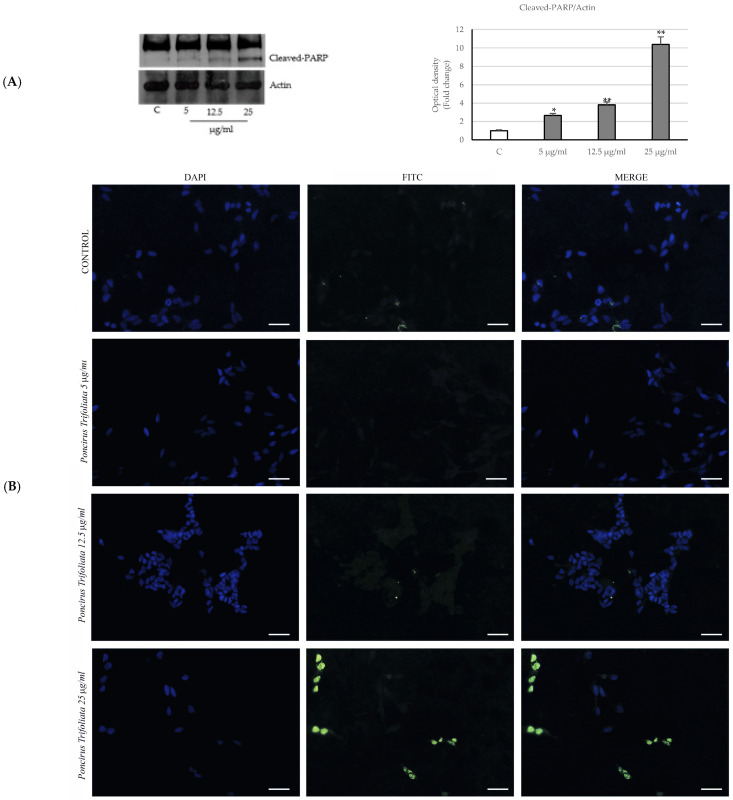
Effects of *P. trifoliata* seed extract on DNA fragmentation. (**A**) Immunoblot of cleaved-PARP levels in LNCaP cells treated or untreated with seed extracts for 24 h. The histogram indicates the optical density of bands normalized for actin (mean ± SD of three experiments). * *p* < 0.05 and ** *p* < 0.001 vs. control (C). (**B**) Terminal deoxynucleotidyl transferase-mediated dUTP nick end labeling (TUNEL) staining in LNCaP cells untreated or treated with *PT* seed extracts for 24 h. DAPI staining was used to visualize the cell nucleus (20× objective).

These events, in treated cells, are also associated with the cleavage of the poly (ADP-ribose) polymerase (PARP) compared to the control (Figure 5A). After that, we determined, by TUNEL assay, whether the treatment was accompanied by fragmentation of the internucleosomal genomic DNA, which is a typical biochemical hallmark of apoptotic cell death. As shown in Figure 5B, the increased percentage of TUNEL-positive cells was revealed particularly in cellular samples treated with 25 µg/mL of *P. trifoliata* extract. Similar findings on the expression of Bcl2 family proteins and PARP proteolytic cleavage were also observed in the PC3 prostate cancer line by *PT* extract (Figure S2).

### 2.4. P. trifoliata Regulates MAPK and mTOR/p70S6K Signaling Pathways in Prostate Cancer Cells

**Figure 6:**
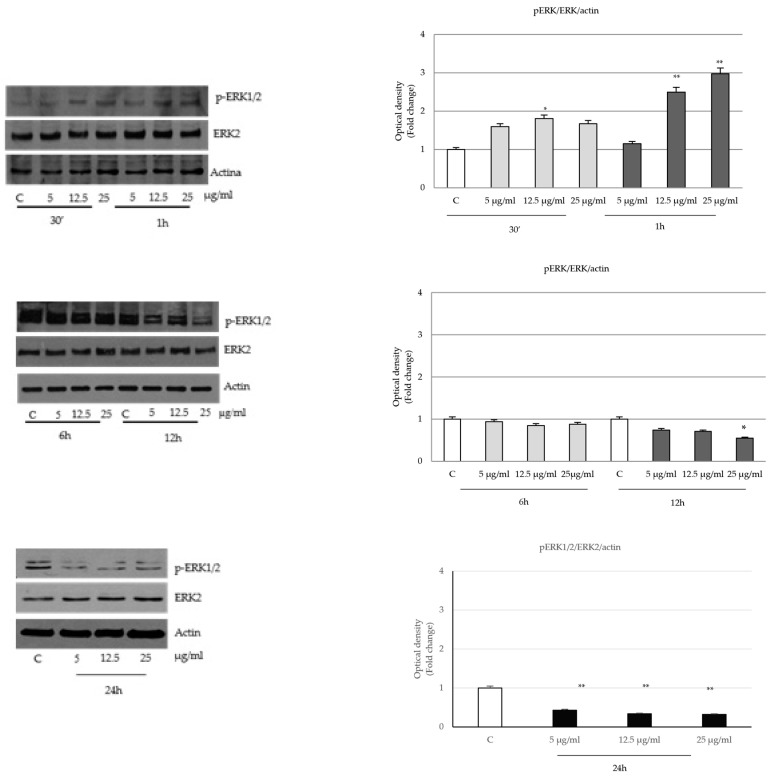
Effects of *P. trifoliata* seed extract on the expression and activation of MAPK family. Immunoblot of ERK1/2 protein phosphorylation levels in LNCaP. The proteins were normalized using actin. Results are presented as the means ± SD of three independent experiments. * *p* < 0.05 and ** *p* < 0.001 vs. control (C).

In different cell lines, the MAPKs family of Ser/Thr protein kinases, including Extracellular Signal-Regulated Kinases (ERK1/2), c-JUN N-terminal kinases (JNKs), and the p38 protein [29], regulates numerous signals, such as transcription, cell death and cell survival, malignant transformation and cell cycle progression [30]. Since the results presented so far have shown a reduction in prostatic cell viability given by the experimental treatment, we wanted to investigate whether *P. trifoliata* seed extract might affect also the MAPK signaling pathways. The results showed that seed extracts in prostatic cells, after a transient activation of phospho ERK 1/2 at the early time of treatment (30 min and 1 h), lead to the inhibition of the same signal starting from 12 h up to 24 h (Figure 6).

**Figure 7:**
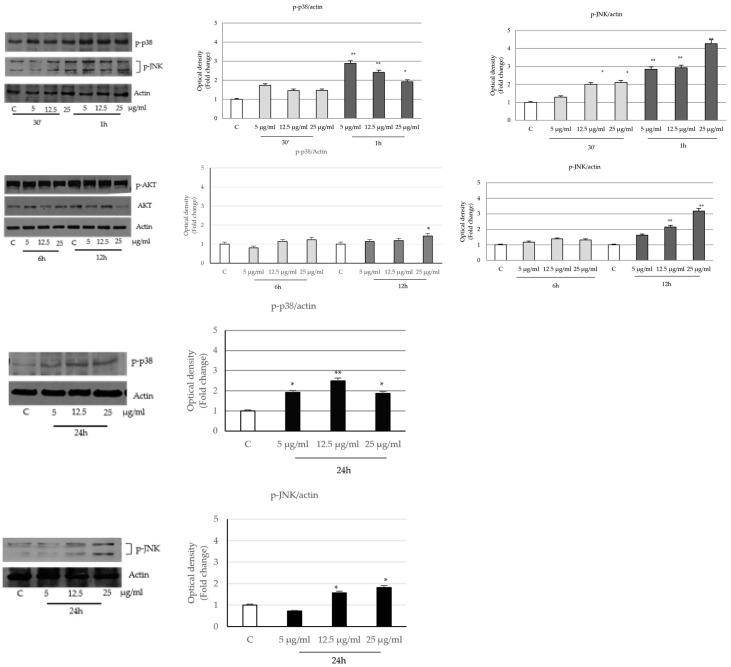
Effects of *P. trifoliata* seed extract on the expression and activation of p38 and JNK MAPK. Immunoblot of p38 and JNK protein phosphorylation levels in LNCaP. Columns are the mean of three independent experiments in which band intensities were evaluated in terms of optical density arbitrary units and expressed as fold over control. * *p* < 0.05 and ** *p* < 0.001 vs. Control (C).

On the contrary, the seed extract promoted the activation of c-Jun NH2-terminal and p38 (mitogen-activated protein) proteins, which remain sustained even at the longest times of treatment (Figure 7).

**Figure 8:**
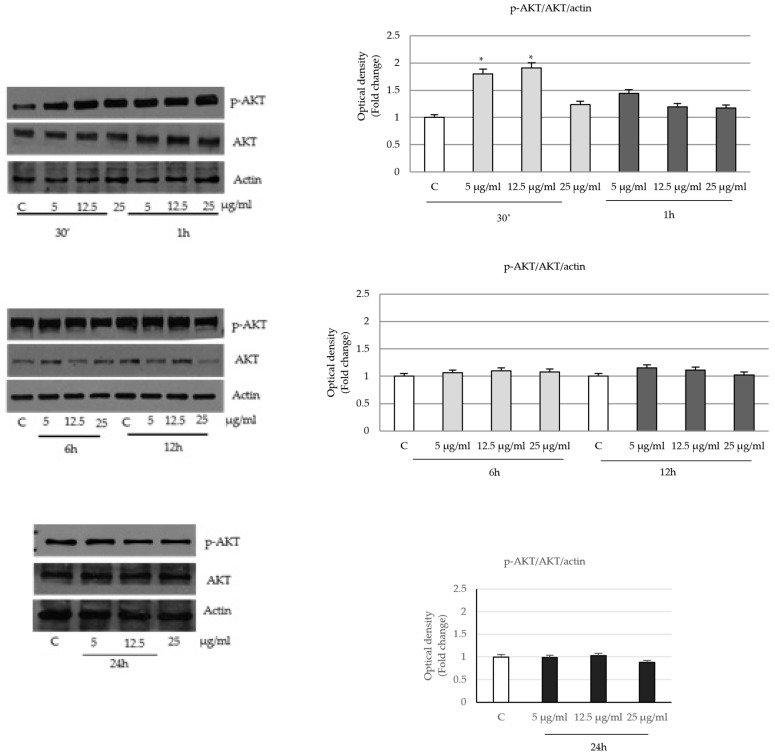
Effects of *P. trifoliata* seed extract on the expression and activation of AKT protein. Immunoblot of AKT protein phosphorylation (ser 473) levels in LNCaP cells untreated or treated with *PT* extract. Actin was used as a protein loading control. The results represent the mean ± SD of three experiments. * *p* < 0.05 vs. Control (C).

Next, we determined whether the *PT* seed extract was able to influence the pro-survival signaling Akt/mTOR kinase. The data showed that after the initial increase in the phospho-Akt levels (ser 473) at 30 min and 1 h, the prolonged treatment over time is able to return the protein signal to values close to the control (Figure 8).

**Figure 9:**
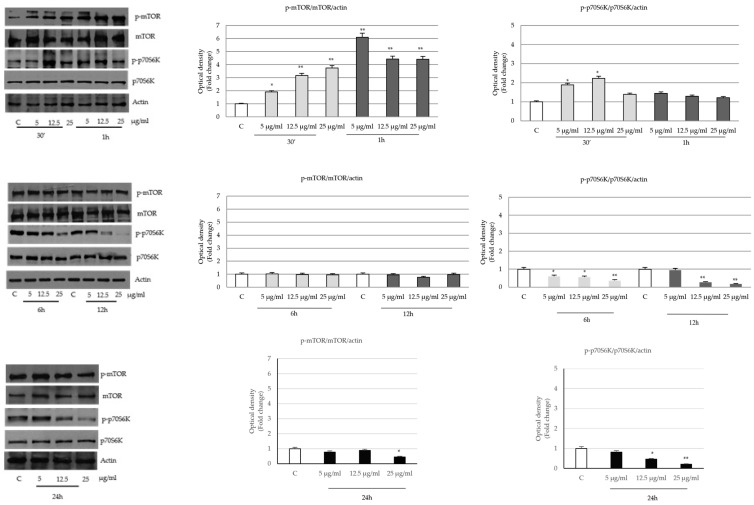
Effects of *P. trifoliata* seed extract on the expression and activation of mTOR and p70S6K proteins. Immunoblot of mTOR and p70S6K protein phosphorylation levels in LNCaP cells untreated or treated with *PT* extract. Histograms represent the mean ± SD of three experiments normalized for actin. * *p* < 0.05 and ** *p* < 0.001 vs. Control (C).

At shorter treatment times, the downstream mTOR (Ser 2448) and p70S6K (Try389) exhibited a pattern similar to p-Akt and, at longer incubation times, they appeared consistently reduced (Figure 9).

## 3. Discussion

Prostate tumor is one of the most common cancers in men [31]. Epidemiological data classify prostate cancer as the second most common type of tumor after lung cancer and the sixth cause of death due to malignant diseases among men worldwide [32].

Numerous epidemiological studies have linked prostatic cancer to a variety of non-modifiable and modifiable factors, including age, ethnicity, family history, food, lifestyle habits and environmental risk exposures. Different evidence in the last decades reported that the androgen receptor is one of the putative factors essential for the growth of the normal and malignant prostate epithelial cells.

The activation of AR drives the expression of PSA, which is a serine protease whose serum levels are used for the diagnosis and monitoring of prostate cancer. Both AR and PSA are involved in the growth and differentiation of the prostate gland, including also all phases of the cancer progression [33]. Furthermore, among the various factors that can promote prostate carcinogenesis, with the transition from less to more aggressive forms, it is important to mention the role of redox homeostasis imbalance and chronic inflammations [34]. In fact, several bioactive natural compounds from fruits and vegetables, due to their antioxidant and anti-inflammatory properties, represent a promising strategy for cancer progression delay. In particular, the highest amounts of bioactive compounds are quantified in peels and seeds [35].

Few previous works have investigated the potential bioactivity of *P. Trifoliata* seed. How and collaborators (2018) demonstrated the ability of *PT* seed extract to suppress the replication of influenza virus, in particular oseltamivir-resistant strains. Interestingly, it was demonstrated that *P. trifoliata* seeds act on the viral endocytosis pathway, while oseltamivir principally inhibits the release of influenza virus by neuraminidase. This mechanism may have important clinical significance [36]. In another study, *P. trifoliata* seeds, together with juice and peel oil, have been demonstrated to possess antioxidant properties and inhibit in vitro α-amylase and α-glucosidase, two key enzymes related to dietary starch digestion in humans, the inhibition of which is one of the best approaches to manage post-prandial hyperglycemia [18].

To the best of our knowledge, no studies have described the anticancer activity of *P. trifoliata* seeds.

*P. trifoliata* is a source of natural compounds, mainly flavonoids, which possesses numerous biological properties, including antioxidant, anti-inflammatory and anti-tumor effects [37,38,39]. In the current research, we have investigated, for the first time in LNCaP prostate cancer cells, the seed extract of *P. trifoliata* that was previously chemically characterized together with its juice by high-performance liquid chromatography with photodiode array detection (HPLC-DAD) [18].

*P. trifoliata* seed extract was characterized by the presence, as dominant constituents, of the flavanones naringin, neohesperedin, and narirutin and the phenolic compound caffeic acid (32.85 µg/g extract) [18]. Previous studies demonstrated that the identified flavanones are able to block the cell cycle and trigger apoptosis in different tumor cell lines [40,41,42]. Naringin and its aglycone naringenin have been demonstrated to be able to suppress through different cellular pathways the growth of several cancer cells. The combination of these flavanones with other chemotherapeutic agents has not only synergistically enhanced the anticancer activity and reduced the side effects of these drugs [40]. In fact, naringin inhibited the cell survival of DU145 cells by inducing cell cycle arrest in the G1 phase and apoptosis through the activation of Bax, p53, Bid, caspase 3, p27^kip1^, p21^cip1^, and cytochrome C and down-regulation of the expression of livin and survivin. In addition, the combination of naringin and paclitaxel synergistically increases the cytotoxic effects of paclitaxel in PC3 and DU145 cells as well as in LNCaP cells. The co-treatment of naringin with docetaxel has almost the same inhibitory effect on cell proliferation in PC3 and DU145 cells but not in LNCaP cells [43]. In different types of tumors, such as lung, pancreatic, thyroid, leukemia and prostatic cancers, narirutin has been shown to inhibit cell growth, induce apoptosis and cell cycle arrest, and reduce inflammation and oxidative stress by acting through different signal transduction pathways, involving PI3K/AKT and MAPK [41].

In human breast adenocarcinoma MDA-MB-231 cells, neohesperidin induced apoptosis by increasing the expression of p53 and Bax and by reducing Bcl-2 protein [42].

Together with these reports, the flavanone glycoside hesperidin in PC3 and DU145 prostate tumor cells inhibited the cell proliferation as well as induced oxidative stress and mitochondrial membrane disruption [44]. Furthermore, the caffeic acid, an additional constituent present in *PT* seed extract, has been found to possess a variety of biological properties, including antioxidant and anti-inflammatory activities [45,46], as well as anticancer effects by decreasing cell migration, invasion and metastases in different tumors, such as breast, lung, melanoma, liver, cervical, colorectal and prostate [47,48,49].

Our results on LNCaP cells proliferation has highlighted a hormetic response of the *PT* seed extract consisting of a modest stimulation at low doses, while the highest concentrations of the extract (up to 12.5 µg/mL) resulted in a significant reduction in cell survival compared to control conditions as well as morphological changes with a decrease in intracellular lipid droplets, which is a marker of energy reserves.

In this study, we demonstrated that the ability of the *P. trifoliata* seed extract to exert the anti-proliferative activity in LNCaP cells occurred through G0/G1 cell cycle arrest due to an up-regulation of the cdk inhibitor p27 protein expression and a reduction in the cyclin D1, which allowed the cells to pass from the G0 phase to synthesis. In fact, the balance between cell division and cell death is regulated through cell cycle checkpoints, which are located in the transition from the G1 to S phase, from the G2 to M phase, and during the S phase [50]. In case of DNA alterations, these “checkpoints”, activated by various internal and external stress factors, interrupt the progression of the cell cycle until the cause of the stress or damage is removed [51].

Cells possess an intrinsic ability to repair damaged DNA, but in the presence of a faulty repair machinery or in the presence of a persistent stressor, damaged cells can be removed through the mechanism of apoptosis. One of the well-known DNA damage-induced downstream effectors is the tumor suppressor p53, which causes cell cycle arrest and apoptosis in target cells [52].

However, in the present study, the block of LNCaP cell cycle given by *P. Trifoliata* seed extract does not involve p53 or p21^waf^ proteins, as they are lowered by the treatment. Rather, it is due to the increase in cdk inhibitor p27 protein and to the down-regulation of phospho-pRb, which is able to maintain, in this latter condition, a proliferative block. The expression of the p27^kip1^ protein is reported to be reduced by the pro-survival Akt kinase through the phosphorylation and inactivation of transcription factors of the AFX/Forkhead family and the inactivation of GSK3β (glycogene synthase kinase 3β) [53,54,55]. In agreement with our data, the lack of Akt activation in LNCaP-treated cells correlates with the increase in p27 levels in order to inhibit the entry into S phase and to direct cells toward the apoptotic response.

Apoptosis exhibits distinct morphological features involving energy-dependent biochemical mechanisms, the activation of caspases, and the functional regulation of specific proteins. The key elements that control apoptotic process are the Bcl-2 family proteins [56]. The members of this family are classified in three subfamilies based on their functions and amino acid sequence similarity. This includes the pro-apoptotic proteins, such as Bim, Bid, and Bad; the pro-survival proteins such as Bcl-2 and Bclxl and the effectors of apoptosis (Bax) [57,58].

Bid, a BH3-domain-only protein, interacts with other proteins of the Bcl-2 family, such as Bax, that oligomerizes and forms pores in the outer mitochondrial membrane, resulting in the release of apoptogenic factors from inside the mitochondria, including cytochrome C, and the consequent activation of effector caspases [57,58]. The antagonistic effects of *PT* seed extract on cell growth and cycle arrest have also been confirmed in the PC3 prostate cancer cell line. In both tumor cell lines, the *P. trifoliata* seed extract addresses cells toward apoptosis by reducing the levels of the anti-apoptotic protein Bcl-2 and increasing the expression of Bax and Bad proteins. In addition, phospho-Bad (Ser112), important to support survival, is reduced in prostatic cells following the treatment with *P. trifoliata* extract.

Another hallmark of apoptosis is the DNA fragmentation that was documented by PARP (poly ADP-ribose polymerase) cleavage and TUNEL assay in LNCaP-treated cells, which was very evident at the highest doses of the phytocomplex. According to these data, studies on *Poncirus* fruit extracts, in different types of cancer cells, have reported anti-tumoral effects consistent with the inhibition of cell proliferation, the induction of mitochondria-mediated apoptosis and with the reduction in cell invasion and migration [59].

In MDA-MB-231 cells, the methanol extract of *P. trifoliata* immature fruits induced apoptosis through the activation of Tumor Necrosis Factor Receptor (TNFR) and TNFR-type I-associated death domain (TRADD) with a consequent increase in caspases. This is linked with the induction of ERK and c-Jun N-terminal kinase (JNK) pathways [60].

Recently, in hepatocellular carcinoma Hep3B and Huh7 cell lines, researchers investigated the ability of the extract obtained from the *P. trifoliata* dried immature fruits to inhibit the invasiveness and migratory capacity of the cells and to induce apoptosis by reducing the Bcl-2 protein levels and by regulating the expression of Bax with a cleavage of caspase-3 and caspase-9 [61].

The arrest of cell proliferation and the activation of programmed cell death led us to analyze MAPK phosphorylative signals that regulate survival and proliferation mechanisms in cancer cells.

MAPK family members such as the kinases ERK1/2 (Extracellular Signal-Regulated Kinases 1 and 2), c-Jun NH2 terminal and p38 (mitogen-activated protein) play an important role in cell growth, differentiation and apoptosis in response to a variety of stress signals and stimuli [62]. The molecular pathways which are mainly involved in the activation of programmed cell death and arrest of cell proliferation are the stress-activated kinase p38 and c-Jun N-terminal kinase MAPK. The p38 MAPK is activated by environmental and genotoxic stresses and by chemotherapeutic agents, such as taxol, doxorubicin, cisplatin and vinblastine [63]. Particularly, JNK modulates the activities of mitochondrial pro- and anti-apoptotic proteins through distinct phosphorylation events, and it activates the intrinsic apoptotic pathway, depending on the cellular context and experimental conditions. In fact, the sustained activation of JNK is associated with apoptosis; conversely, fast and transient activation is associated with cell survival events [64,65].

More generally, ERKs support cell survival and possess anti-apoptotic activity, although they, in some conditions, have also been involved in mediating chemotherapy-induced apoptosis [66,67,68].

Our data indicate that *P. trifoliata* extract increases the levels of MAPK stress stimuli, such as p38 and JNK in LNCaP cells, while it reduces the survival signaling sustained by ERK1/2, mTOR and p70s6k. The different action on the kinase pathways explains the inhibitory activities of *PT* seed extract on cell proliferation and apoptosis.

Oncogenic activation of the phosphatidylinositol-3-kinase (PI3K), protein kinase B (PKB/AKT), and mammalian target of rapamycin (mTOR) pathway is a frequent event in prostate cancer that facilitates tumor formation, disease progression and therapeutic resistance. Likewise, the AKT activation is also involved in prostate cancer cell migration and survival [69].

Our results have reported that in *P. trifoliata*-treated cells, the pro-survival pAKT is not down-regulated as expected. In fact, the phosphorylative signal remains well expressed, showing a transient activation at short treatment times, and then it is kept stable with the length of the treatment time. This observation suggests that the anticancer effect of the *Poncirus* seed extract in prostatic cells does not target and influence pAKT but still affects other downstream signals, such as mTOR and p70s6k.

The activation of p70S6K through phosphorylation on multiple Ser/Thr residues mediated by mTOR, in response to nutrients and growth factors, plays an essential role in tumor growth and metastasis. In fact, the kinase is able to sustain ribosomal activity, protein biosynthesis and energetic metabolism [70,71,72]. Therefore, the down-regulation of the mTOR/P70S6K signaling could be a potential approach to counteract prostatic tumor growth and progression as evidenced in this study by the long treatment of LNCaP cells with *P. trifoliata* seeds extract. In addition, we have shown that the treatment with phytocomplex induces a reduction in AR and PSA in LNCaP cells, which is coincident with the decrease in other cell survival signals. Specifically, it has been reported that AR activation controls the transition from the G1 to S phase of the cell cycle by increasing the expression of cyclin D1, which in turn promotes cell cycle progression through the activation of CDK4/6 cyclin-dependent kinases [73]. Taken together, this study demonstrated, for the first time, that *P. trifoliata* seed extract treatment inhibited cell viability and induced apoptosis in LNCaP cells. In addition, we also showed that ERK1/2 MAPK and mTOR/P70S6K pathways, which are essential for carcinogenesis and the progression of prostate cancer, was inhibited by *P. trifoliata* extract. Therefore, on the basis of these findings, the future directions will be to identify which single component of the *PT* seed extract detains the best anti-proliferative and pro-apoptotic properties.

This could be advantageous to implement the use of one or more of these components in the adjuvant therapy of prostate cancer.

## 4. Materials and Methods

### 4.1. Plant Materials

The fruits of *P. trifoliata* were collected in the Botanic Garden of the University of Calabria (Italy) and identified by Dr. N.G. Passalacqua, University of Calabria. Fruits were examined for integrity and the absence of dust and insect contamination.

### 4.2. Extraction Procedure and Chemical Profile of P. trifoliata Seed Extract

The seeds of *P. trifoliata* (657 g) were exhaustively extracted by maceration using methanol as solvent (5 × 700 mL). The extract solutions were filtered and dried under vacuum. The resulting dried *P. trifoliata* seed extract (PT) was stored in a dark-brown glass at 4 °C for further analysis. As previously reported, the extract was chemically characterized by high-performance liquid chromatography–diode array detection (HPLC-DAD) by using a Knauer (Asi Advanced Scientific Instruments, Berlin, Germany) system equipped with two pumps (Smartiline Pump 1000, SpectraLab Scientific Inc., Markham, ON, Canada), a Rheodyne injection valve (20 μL) and a photodiode array detector UV/VIS [18]. Water/formic acid (99.9:0.1, *v*/*v*; solvent A) and acetonitrile/formic acid (99.9:0.1, *v*/*v*; solvent B) were used as the mobile phase. The gradient profile was 0.01–20.00 min, 5% B isocratic; 20.01–50.00 min, 5–40% B; 50.01–55.00 min, 40–95% B; 55.01–60.00 min, 95% B isocratic. The flow rate was 1.0 mL/min. Peaks were monitored at 280 and 350 nm. Identification and quantification were carried out based on recorded retention times in comparison with authentic standards. Analyses were performed in triplicate.

### 4.3. Cell Culture

LNCaP and PC3 cells were obtained from the American Type Culture Collection (ATCC) and were grown in RPMI 1640 medium supplemented with L-glutamine, penicillin G and 10% fetal bovine serum (FBS) (Life Technologies, Monza, Italy) at 37 °C in a humidified atmosphere containing 95% air and 5% CO_2_. Subconfluent cell culture, synchronized for 24 h in RPMI without phenol red and serum (PRF-SFM RPMI), was used for all experiments. All experiments were performed with mycoplasma-free cells (Applied Biological Materials Inc., Vancouver, BC, Canada).

### 4.4. Cell Viability Assay

LNCaP and PC3 cells, after the attachment in 96-well plates, were treated with the increasing concentrations of seed extract for 24 and 48 h. Next, in each well, 100 µL of 2 mg/mL MTT (3-[4,5-dimethylthiazol-2-yl]-2,5-diphenyl tetrazolium) (Sigma-Aldrich, Merck, Darmstadt, Germany) was added for 4 h at 37 °C. Finally, after removing the MTT, 100 μL/well DMSO was added to solubilize the formazan, and the plates were read at 570 nm with a plate reader (Multiskan EX, Thermofisher System, Waltham, MA, USA).

### 4.5. Flow Cytometer Analysis

At the end of treatments, LNCaP and PC3 cells were washed with PBS and fixed in methanol (50%) overnight at −20 °C. Next, the prostatic cells were stained with a solution containing: propidium iodide (50 µg/mL), RNAse-A (20 U/mL) and Triton (0.1%) (Merck Life Science, Milan, Italy). The DNA content was measured using a FACScan flow cytometer (Becton Dickinson, Mountain View, CA, USA), and the data were acquired using CellQuest software (version 4.0). Cell cycle profiles were determined using ModFit LT [74].

### 4.6. Immunoblotting Analysis

After the treatments, LNCaP and PC3 cells were lysed with 500 μL of RIPA buffer containing the following: Tris-HCl (50 mM), NaCl (150 mM), NP-40 (1%), sodium deoxycholate (0.5%), sodium fluoride (2 mM), EDTA (2 mM), SDS (0.1) and protease inhibitors: aprotinin (1.7 mg/mL), leupeptin (1 mg/mL), phenylmethylsulfonyl fluoride (200 mmol/L), sodium orthovanadate (200 mmol/L) and sodium fluoride (100 mmol/L); (Sigma-Aldrich, Merck).

The proteins, after the electrophoretic run on 8% and 12% SDS/polyacrylamide gel, were transferred to a nitrocellulose membrane. Next, the membranes were probed with primary antibodies against the following proteins: cyclin D1, p-JNK, JNK, p-p38, and p-38 (Invitrogen, Thermo Fisher Scientific); Bax, Bcl2, Bad, p-Bad, ERK2, p53, p27, p21, p-Rb, Rb and actin (Santa Cruz Biotechnology, DBA, Milan, Italy); and p-ERK, pAKT and AKT (Cell Signaling Technology, Euroclone, Milan, Italy).

At the end of incubation, the membranes were washed and probed with peroxidase-coupled goat anti-mouse or anti-rabbit antibodies. Finally, the antigen–antibody complex was revealed using the chemiluminescent substrate for Western blotting, ECL System (Amersham Pharmacia, Buckinghamshire, UK) [74].

### 4.7. Immunocytochemical Staining

For morphological analysis, we used May–Grumwald–Giemsa staining. Briefly, cells treated for 24 h with seed extracts were fixed in methanol for 5–10 min. After fixation, cells were stained with May–Grunwald working solution for 10 min and subsequently washed in pH 6.8 buffer. After rinsing, cells were stained with diluted Giemsa stain for 30 min. After that, cells were washed with distilled water and allowed to dry. Then, cells were analyzed and photographed by using the optical microscope (original magnification, ×200).

### 4.8. The Lipid Oil Red O Staining

LNCaP-treated cells, after washing with PBS, were fixed on glass coverslips for 48 h with 4% paraformaldehyde for 30 min at RT. Subsequently, 0.5% Oil Red O (Sigma-Aldrich, St. Louis, MO, USA) solution was used to stain cells for 20 min at RT and counterstained with hematoxylin for 2 min. Finally, the cells were washed with PBS and observed under the microscope (scale bars 12.5 µm).

### 4.9. TUNEL Assay for Apoptosis Detection

An APO-BrdUTM TUNEL Assay Kit (Promega) was used to analyze TUNEL labeling. LNCaP cells, after fixation in 4% paraformaldehyde solution in PBS (pH 7.4) for 25 min at 4 °C, were permeabilized with PBS and Triton (0.2%) solution for 5 min. Next, the cells were washed twice with washing buffer for 5 min and recovered with 100 µL of equilibration buffer at room temperature for 5–10 min. Terminal deoxynucleotidyl transferase end-labeling TdT and fluorescein-dUTP cocktail was used for each sample and incubated for 1 h at 37 °C. Specifically, in the nicked DNA, TdT catalyzes the binding of fluorescein-dUTP to free 3′ OH ends. Then, after washing with 20× SSC solution buffer, to stain nuclei, the LNCaP cells were incubated with 100 µL DAPI (4′,6-diamidino-2-phenylindole). Finally, the cells were observed and photographed using a fluorescent microscope (20× objective).

### 4.10. Statistical Data Analysis

The results obtained from different and independent experiments were expressed as the mean ± standard deviation (SD). For statistical significance, the analysis of the data was completed using the Bonferroni post-test. Moreover, the significant differences between two groups of samples were performed by Student’s *t*-test for unpaired data (2-tailed). Differences were considered significant when *p* ≤ 0.05 and *p* ≤ 0.001. The GraphPad Prism software 4 was used for statistical tests. (GraphPad Software, La Jolla, CA, USA).

## Figures and Tables

**Table 1:** Identified constituents of *P. trifoliata* seed extract [18].

Compound	μg/g Extract
*Flavanone-O-glycosides*	
Narirutin	37.62 ± 0.53
Naringin	156.42 ± 0.35
Neohesperidin	80.12 ± 0.25
*Phenolic acid*	
Caffeic acid	32.85 ± 0.11

Data are expressed as mean ± standard deviation (*n* = 3).

## Data Availability

No new data were created or analyzed in this study. Data sharing is not applicable to this article.

## Supplementary Materials

The following supporting information can be downloaded at https://www.mdpi.com/article/10.3390/ijms242216351/s1.
