# Supplementary material for: Poncirus trifoliata (L.) Raf. Seed Extract Induces Cell Cycle Arrest and Apoptosis in the Androgen Receptor Positive LNCaP Prostate Cancer Cells
Source: Int J Mol Sci. 2023 Nov 15;24(22):16351. doi: 10.3390/ijms242216351 (PMC10671002; doi:10.3390/ijms242216351)
Supplement: Supplementary file 1 [file ijms-24-16351-s001.zip › ijms-2616309-supplementary.pdf]

A

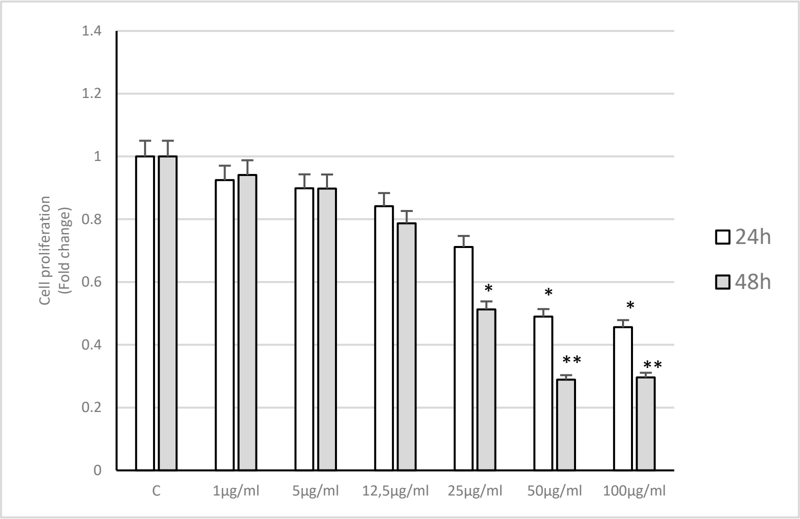

B

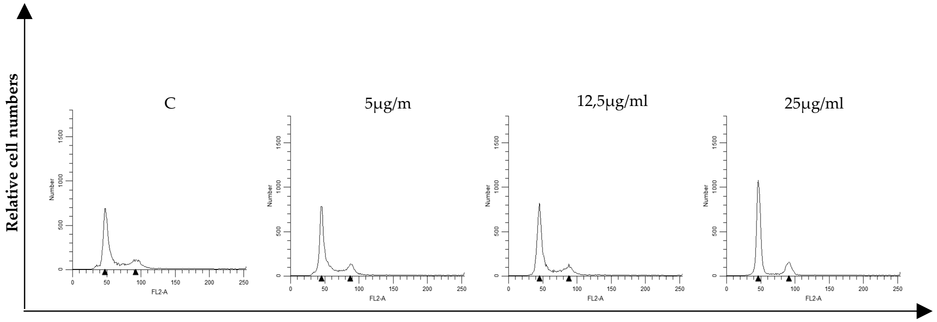

C

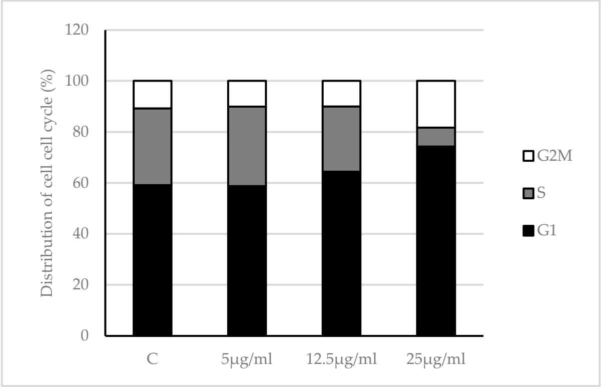

Figure S1 *PT* seed extracts-induced inhibition of proliferation and arrest of the cell cycle. A) PC3 cells were treated with increasing doses (1, 5, 12,5, 25, 50 and 100µg/ml) of *PT* seed extracts for 24 and 48h. Cell proliferation is expressed as fold change +- S.D. relative to control (C) cells and is representative of three different experiments, each performed in triplicate. *p≤0.05 and **p≤0.001.
B) PC3 cells were treated with various concentrations of *PT* seed extracts for 24h, stained with propidium iodide (PI) and analyzed on a FACScan Flow cytometer.
C) Quantitative analysis of percentage gated cells at G0/G1, S and G2/M phases were shown.

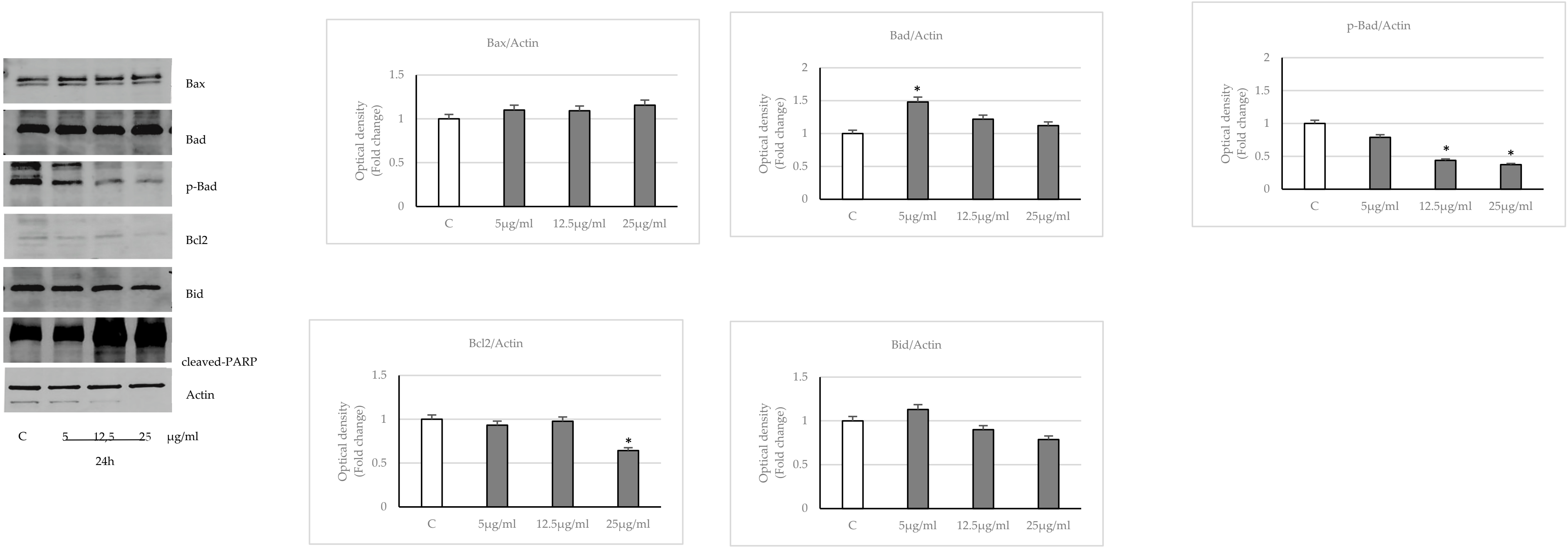

Figure S2: Effects of *P. trifoliata* seed extracts on the expression of apoptosis proteins. Immunoblotting of Bax, Bad, p-Bad, Bcl2, Bid and cleaved-PARP expression levels in PC3 cells untreated or treated with extract for 24h. The actin is used as loading control. Histograms represent the mean SD of three experiments in which band intensities were evaluated in terms of arbitrary units of optical density and expressed as fold change relative to control. *p≤0.05 vs Control (C).

Comp	LNCaP		PC3	
P. trifoliata seed extract	IC_{50}	95% CI	IC_{50}	95% CI
	22.98	18.68 – 26.97	25.85	23.09 – 29.18

Table S1. IC_{50} in LNCaP and PC3 cell lines. IC_{50} of *Poncirus trifoliata* seed extract for LNCaP and PC3 prostate cancer cells on anchorage-dependent growth. Abbreviations: IC_{50}, half-maximal inhibitory concentration; CI, Confidence Interval.
